# Street-level diplomacy and local enforcement for meat safety in northern Tanzania: knowledge, pragmatism and trust

**DOI:** 10.1186/s12889-019-7067-8

**Published:** 2019-07-03

**Authors:** T. A. Hrynick, V. Barasa, J. Benschop, S. Cleaveland, J. A. Crump, M. Davis, B. Mariki, B. T. Mmbaga, N. Mtui-Malamsha, G. Prinsen, J. Sharp, E. Sindiyo, E. S. Swai, K. M. Thomas, R. Zadoks, L. Waldman

**Affiliations:** 1 Institute of Development Studies, University of Sussex, Brighton, UK; 2 mEpiLab, School of Veterinary Science, Massey University, Palmerston, New Zealand; 3 Institute of Biodiversity, Animal Health and Comparative Medicine, College of Medical, Veterinary and Life Sciences, University of Glasgow, Glasgow, UK; 4 Nelson Mandela African Institute of Science and Technology, Arusha, Tanzania; 5 Centre for International Health, Dunedin School of Medicine, University of Otago, Dunedin, New Zealand; 6 Kilimanjaro Christian Medical University College, Tumaini University, Moshi, Tanzania; 7 Paul G. Allen School for Global Animal Health, College of Veterinary Medicine, Washington State University, Pullman, USA; 8 Tanzania Chamber of Commerce - Kilimanjaro, Moshi, Tanzania; 9 Kilimanjaro Clinical Research Institute, Moshi, Tanzania; 10 Ministry of Livestock and Fisheries Development, Dodoma, Tanzania; 11 School of People, Environment and Planning, Massey University, Palmerston North, New Zealand; 12 School of Geographical and Earth Sciences, University of Glasgow, Glasgow, UK; 13 Mwanga District Council Department of Livestock and Fisheries, Mwanga, Tanzania

**Keywords:** Butchers, Extension officers, Food safety, Frontline actors, Meat safety, Policy implementation, Tanzania

## Abstract

**Background:**

With increasing demand for red meat in Tanzania comes heightened potential for zoonotic infections in animals and humans that disproportionately affect poor communities. A range of frontline government employees work to protect public health, providing services for people engaged in animal-based livelihoods (livestock owners and butchers), and enforcing meat safety and food premises standards. In contrast to literature which emphasises the inadequacy of extension support and food safety policy implementation in low- and middle-income countries, this paper foregrounds the ‘street-level diplomacy’ deployed by frontline actors operating in challenging contexts.

**Methods:**

This research is based on semi-structured interviews with 61 government employees, including livestock extension officers/meat inspectors and health officers, across 10 randomly-selected rural and urban wards.

**Results:**

Frontline actors combined formal and informal strategies including the leveraging of formal policy texts and relationships with other state employees, remaining flexible and recognising that poverty constrained people’s ability to comply with health regulations. They emphasised the need to work with livestock keepers and butchers to build their knowledge to self-regulate and to work collaboratively to ensure meat safety. Remaining adaptive and being hesitant to act punitively unless absolutely necessary cultivated trust and positive relations, making those engaged in animal-based livelihoods more open to learning from and cooperating with extension officers and inspectors. This may result in higher levels of meat safety than might be the case if frontline actors stringently enforced regulations.

**Conclusion:**

The current tendency to view frontline actors’ partial enforcement of meat safety regulations as a failure obscures the creative and proactive ways in which they seek to ensure meat safety in a context of limited resources. Their application of ‘street-level diplomacy’ enables them to be sensitive to local socio-economic realities, to respect local social norms and expectations and to build support for health safety interventions when necessary. More explicitly acknowledging the role of trust and positive state-society relations and the diplomatic skills deployed by frontline actors as a formal part of their inspection duties offers new perspectives and enhanced understandings on the complicated nature of their work and what might be done to support them.

**Electronic supplementary material:**

The online version of this article (10.1186/s12889-019-7067-8) contains supplementary material, which is available to authorized users.

## Background

The growing scale and quickening pace of economic, political, social and ecological change around the world has increased the risk of zoonotic pathogen emergence and re-emergence, and of associated outbreaks of disease in animals and humans [1, 2]. Interrelated processes of globalisation, population growth, urbanisation, climate change, conflict, shifting land use and disruption of traditional socio-environmental systems, changing diets and livelihoods are bringing people, animals and microbes into novel interactions creating new, complex and dynamic channels of disease risk, especially in low- and middle-income countries (LMICs) [3–6].

Tanzania – in addition to undergoing many of the aforementioned processes – hosts Africa’s third largest concentration of livestock and, with its abundant biodiversity, is vulnerable to emerging, re-emerging and endemic zoonoses [7–9]. Outbreak events such as the 2006/2007 Rift Valley Fever (RVF) epidemic have wrought substantial economic, social and psychological damage, tending to primarily affect marginalised populations including poor urban and peri-urban dwellers, and rural, pastoral and agro-pastoral people [10, 11]. While outbreaks of some zoonoses, such as RVF, Ebola, anthrax, Severe Acute Respiratory Syndrome (SARS), Middle East Respiratory Syndrome (MERS), and avian and swine influenza garner attention and concern, a number of endemic zoonoses – such as brucellosis, leptospirosis, Q fever and non-typhoidal *Salmonella* - attract far less consideration and yet have important if less newsworthy, negative consequences for individual, community and national development in Tanzania and across the global south [12–15].

One potential way humans can become infected with zoonotic pathogens is through animal product consumption, including red meat [16, 17]. Rural, poor and predominantly small-scale farmers undertake the majority of Tanzania’s livestock production [18]. Here, growing urban populations and rising incomes have increased meat demand [19]. Beef is favoured, and, in 2017, accounted for 82% of meat production [20]. Overall, Tanzanians prefer fresh produce – including freshly butchered ‘warm’ red meat – which they perceive to be locally or domestically sourced and the vast majority of people purchase meat in small, local butcheries on days they intend to cook and consume it [19, 21, 22].

Meat value chain actors in Tanzania include livestock farmers, handlers, traders, transporters, processors, owners of slaughter sites, butcheries, eateries and associated workers. They are subject to regulations governing how animals are cared for, transported, slaughtered, processed, stored and sold, which include procedural, infrastructural and personnel standards. Considerable national-level legislation exists to inform management of animal health, slaughter and meat sale including the Animal Disease Act (2003); Tanzania Food, Drugs and Cosmetics Act (2003); Public Health Act (2009); Standards Act (2009); Meat Industry Act (2006); Veterinary Act (2003) and others. National oversight of food safety lies with multiple state bodies including the Tanzania Food and Drugs Authority, the Ministry of Livestock and Fisheries, and the Tanzanian Bureau of Standards [19] which are responsible for technical regulations. Ultimate responsibility for monitoring, inspection and animal health service delivery however, is decentralised and is the remit of Local Government Authorities at district, ward and village level [23, 24]. So long as local provision remains within the bounds set by national law and regulations, standards may vary as local decision-making and bylaws drawn up by LGAs manage issues in locally appropriate and acceptable ways.

While a range of Tanzanian government actors are responsible for ensuring meat is safe for human consumption, monitoring regulatory compliance, and ensuring disease events in animals and humans are prevented or quickly stopped, those most central to this at ward and village level are public Health Officers (HOs) and Livestock Extension Officers (LEOs), many of whom also work as meat inspectors [25].^1^ LEOs’ and HOs’ responsibilities, in relation to the prevention and mitigation of zoonotic disease in animals and related meat-borne illness, fall into three overlapping activities, namely: preventative measures and animal-based livelihood support; the management of disease incidents and outbreaks; and meat site (slaughter sites, butcheries, other meat retailers) inspection, monitoring, and related sanction.

In many countries, the implementation of government policy and regulation, and extension officers’ work has been recognised as challenging and often inadequate [21, 25, 26]. Theoretical policy implementation literature shows, in keeping with this, that this is seldom unproblematic, with frequent ‘implementation failures’ [27, 28]. In seeking to understand these failures, attention has focused on how administrative directives and processes interact with contextual, socio-economic, cultural and political aspects to shape implementation. Bringing the top-down, ‘highly scripted’ dimension of policy implementation together with its bottom-up, informal dimensions has introduced new emphasis on governance arrangements, policy networks, and institutional relationships [27]. The space between these top-down and bottom-up dimensions is occupied by frontline actors, or ‘street-level bureaucrats’ [29], who have degrees of ‘decisional latitude’ to determine which policy directives they respond to and how they satisfy government objectives while accommodating local, contextual factors, and retaining professional autonomy [27, 30, 31]. These frontline actors bring their own values, knowledge, and norms to bear as they enter into processes of ‘negotiation and bargaining’ with people who form the policy target [32]. Despite widespread recognition of frontline actors’ discretionary roles in mediating ‘technically complex policy matters’, the ways in which they undertake policy implementation remain ‘opaque’ [31], especially in LMICs.

While much research has focused on how frontline actors’ discretion might result in policies being operationalised in ways that contradict or undermine policy makers’ intentions [29, 30], far less attention has been paid to the skills required to implement policy, particularly in contexts of limited resources. The emphasis has been on frontline actors’ ability to subvert and reorient policy through implementation, rather than exploring how their flexibility and discretionary powers can be used to implement policy, or approximate policy goals even if through unprescribed or unorthodox strategies. This paper explores the day-to-day strategies employed by frontline actors such as LEOs and HOs to do their work in relation to meat safety in northern Tanzania. Taking an actor-oriented approach [33], it distinguishes itself from the bulk of literature on the meat value chain and food and meat safety policy implementation in LMICs which emphasises deficiencies and inadequacies of the food safety system and associated technical staff [24, 34, 35]. Instead, drawing on policy implementation literature and the concept of ‘street-level diplomacy’ which emphasises relationships, interpersonal trust and the use of negotiation to enhance policy compliance [28, 36, 37], this paper foregrounds the experiences, perceptions, knowledge and ‘soft’ skills of staff to understand how they do their jobs and why they choose to operate in particular ways. Through this, it explores how creative day-to-day enactment of health and safety can, and needs to be, recognised in contexts where policy may be regarded as appropriate, yet resources for implementation remain scarce and demonstrates the importance of social inquiry to understanding and tackling disease in LMICs [38].

## Methods

This paper is based on 61 semi-structured interviews conducted with regional (Kilimanjaro) (*n* = 2), district (Moshi Municipal) (*n* = 4), and ward-level (*n* = 55) administrators and technical staff with general, human and/or animal health mandates; elected ward-level councillors; and ward-level health committee volunteers in order to capture a wide range of actors and activities in local governance and management of zoonotic disease and meat safety. In this paper, the concept of ‘meat safety’ is used to refer to the presence or absence of pathogens in meat which can cause disease in humans [39]. This recognises that the presence/absence of pathogens in meat is not independent from livestock health and its management; nor of how meat is handled or its handlers regulated, and acknowledges the social/political interfaces between state actors and private citizens. Regional and district-level actors were selected for their key roles, while those filling positions of interest in each of five randomly selected wards from Moshi Rural District and five from Moshi Municipality, were also interviewed (see Table 1). This emphasis on a wide range of respondents was informed by the One Health framework which recognises the connections between animal, human and environmental health [40]. In most cases, each relevant position was occupied by one individual in each respective ward.^2^ In some instances, the position was either not occupied or the position-holder declined to be interviewed. In another, one person occupied two roles of interest. This meant a total of 55 ward-level respondents were identified and interviewed. A further six key informants were identified at regional and district levels, bringing the total to 61. Because decisions about whom to interview were made in relation to particular posts, gender and other social markers were not considered in the selection process. The disproportionately low number of female interviewees in livestock extension/meat inspection and managerial/leadership roles reflects socio-cultural associations between masculinity, livestock-rearing and red meat [41]^3^ and broader, widely-observed gendered power relations and barriers to African women’s professional advancement [42–44].

**Table 1:** Interviewees’ role, location and gender

	Respondent roles	Moshi rural district (rural, peri-urban)	Moshi municipality (urban)	Regional
Frontline technical staff	Livestock Field Officers (LFOs/LEOs)	5 (M = 4, F = 1)	5 (M = 3, F = 2)	-
	Public Health Officers (HOs)	4^a^ (M = 1, F = 3)	5^b^ (M = 1, F = 4)	-
	Health & Environment Committee volunteers	5 (M = 2, F = 3)	5 (M = 2, F = 3)	-
	Clinic-based human medical workers	5 (M = 1, F = 4)	4^a^ (M = 2, F = 2)	-
	**Subtotal**	**19 (M = 8, F = 11)**	**19 (M = 8, F = 11)**	-
Administrative & elected officials	Ward Executive Officers	5 (M = 4, F = 1)	5^b^ (M = 3, F = 2)	-
	Elected ward-level officials	5 (M = 5)	3^a^ (M = 3)	-
	High-ranking municipal official	-	1 (M = 1)	-
	District-level veterinary operatives	-	3 (M = 3)	-
	Regional-level animal health operatives	-	-	2 (M = 2)
	**Subtotal**	**10 (M = 9, F = 1)**	**12 (M = 10, F = 2)**	**2 (M = 2)**
	**Total respondents**	**61 (M = 36, F = 25)**		

*M* Male, *F* Female

^a^Indicates that one or more wards either did not have an individual in post at the time, or he/she was not available for interview

^b^One respondent held the position of both Ward Executive Officer and HO for his ward

The subtotals are presented in bold

Interviews took place in situ from February 2017 to February 2018 in Kiswahili before being translated and transcribed into English by the author (BM). Back translation into Kiswahili was deemed unnecessary as the interviewer (BM) was involved in transcription, translation and reviewing interpretations and meanings used in this paper. Moreover, this research prioritises situated knowledge produced through interactions between respondents and the interviewer in relatively open-ended dialogue rather than literal objective translation, or consistent sets of meaning [45].

As this research took an inductive approach, open-ended, semi-structured interview schedules were designed – in consultation with a number of experts on the topic, the interviewer, and senior Tanzanian policy makers – to guide discussion and to elicit contextually-rich pictures of the roles, routines, experiences, challenges, strategies, and perceptions of respondents in relation to policy, meat safety, animal health and zoonoses.^4^

Data analysis was approached inductively and conducted through a grounded approach, allowing conceptual and theoretical insights to emerge from the data rather than from preconceived notions derived from existing literature. Interview data were first thoroughly read by the two lead authors (TH and LW) to gain a holistic sense of themes, patterns and relationships [46]. Open coding techniques were used and a preliminary coding structure developed. Then, through several cycles of reading, rereading and coding the data using Nvivo 12 (QSR International, Australia), the coding structure evolved, expanding and transforming as increased familiarity with the data yielded new insights, relationships, categories and abstractions [46, 47]. Through this process of ‘emergent flexibility’ [48], we noted interesting patterns pointing to what we came to interpret as ‘diplomacy’ operating at the ‘street’ level, leading to the observations, assertions and links to existing literature underpinning this paper.

Respondents not directly involved in meat inspection, livestock, and environmental and public health enforcement (elected politicians, general administrators and supervisors, human clinical workers) discussed their roles in relation to, or signalled the importance of LEOs and HOs, of which we interviewed ten and nine respectively. The centrality of these workers as frontline service providers, inspectors and law enforcers led us to focus primarily on them. Working under the administrative supervision of Ward Executive Officers, and technical supervision of their district-level superiors, their primary responsibilities fall into three overlapping categories.

The first set of activities, disease preventative measures, involves LEOs dispensing animal husbandry advice to livestock keepers, occasional treatment of livestock, and carrying out annual livestock vaccination campaigns against diseases of importance, as determined by district authorities, usually anthrax and rabies. LEOs mentioned they carried out routine visits to livestock keepers on a daily basis, suggesting at times that these visits were both solicited and routine. Appropriately-qualified LEOs are allowed, but not officially required, to provide vaccines and other drugs, and many use their own money to purchase them from private shops to then sell to livestock keepers [25, 49]. Although historical government subsidization of animal drugs ceased in the 1990s in favour of a private market system, occasionally vaccines are free or subsidized, usually in response to outbreaks or through donor-funded initiatives [50].

The second set of activities involves the management of serious animal or related human disease, whether a singular occurrence, or outbreak. For serious diseases, such as anthrax, this may include emergency vaccination, treatment of humans and animals, quarantine, and safe disposal or condemnation of infected meat, carcasses or animals [51]. Given there is no formal surveillance of foodborne disease in Tanzania however [52], many less spectacular disease incidents remain undetected, un-investigated and unreported.

**Fig. 1:**
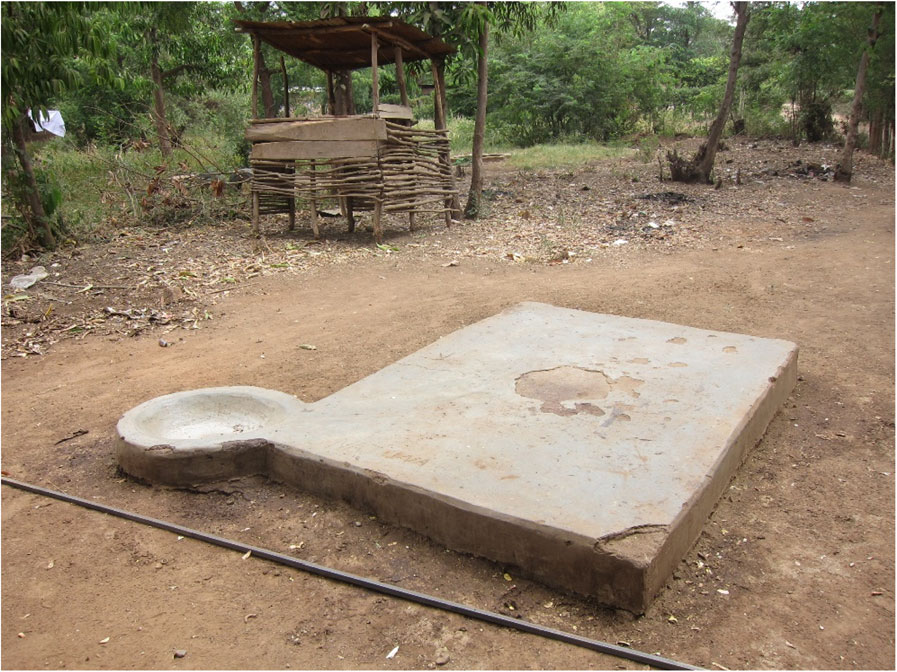
- Slaughter slab in a rural ward (photograph by HAZEL Consortium member)

The third set of activities involves monitoring and inspection of meat, and the establishments it passes through in order to protect consumers and prevent zoonotic disease transmission. Many LEOs perform meat inspection (ante- and post-mortem) at small slaughter slabs – often simple cement platforms of three or four square meters, usually owned by individual butchers and often adjacent to their butcheries (see Fig. 1) – or at larger more centralised publicly- or privately-owned slaughterhouses.^5^ Inspection involves visual assessment, palpation, and muscle and organ incision with additional detailed carcass examination if organ lesions are detected, and subsequent condemnation of meat/organs unfit for consumption [53, 54].

LEOs should also prevent and stop sales of uninspected meat, inspected but condemned meat and unauthorised livestock slaughter. They mentioned making follow-up visits, often reported as random or ad-hoc, to butcheries to ensure meat for sale has been inspected and marked safe with a government stamp, and that workers and butchery facilities meet required standards, although the implied frequency with which they carried out such visits varied from ‘occasionally’ to daily and duration of these visits was not commented on. Standards which respondents emphasised included that workers wear uniforms and have clean bills of health and that butcheries are outfitted with tiled walls, plastic chopping boards, electric or manual meat saws, running water and screens^6^ to protect meat from flies and contaminants. HOs also inspect butcheries and eateries for health and hygiene standards, checking that premises are generally clean and that staff have current medical certificates. While no respondents mentioned enforcing standards relating to slaughter process, government documents advocate particular methods, such as regular sterilization of knives, and careful isolation of gut contents [53]. When encountering non-compliance – unstamped meat for sale, or butcheries without handwashing facilities for instance – LEOs and HOs are responsible for meting out sanctions such as fines, condemning meat or closing offending businesses.

## Results

### Challenges for animal health and meat safety

Tanzania has committed to a One Health agenda to acknowledge and act upon linkages between animal, human, and environmental health, and this includes recognition of foodborne and meat-borne disease [24, 55, 56]. This has resulted in greater support and collaboration for integrated research, including on the biosocial dimensions of zoonoses [40] but it is as yet unclear how this has affected frontline actors’ daily activities to promote livestock health and prevent foodborne illness. The veterinary sector in Tanzania, as in other sub-Saharan countries, lacks capacity for service provision and enforcement, and struggles with inadequate investment [20, 38, 49]. Only 20% of Tanzania’s rural livestock keepers utilise extension services [18] and only 6% of the country’s approximately 12 thousand registered villages have village-level LEOs. These low numbers result, in part, from recruitment cuts beginning in the mid-1990s, and in part, from bureaucratic reforms to privatise veterinary services in spite of livestock keepers’ inability to pay for them and poor rural infrastructure [49, 50]. Consequentially, state LEOs are often the only livestock professionals to which communities – and particularly rural ones – have access, although there may be other informal actors such as community animal health workers [25, 57, 58].

LEOs, and particularly those assigned to rural wards, are often charged with covering large geographical areas, and many lack official, or adequate transportation [25]. This, along with few colleagues with whom to share responsibility, was the most frequently cited challenge from both urban and rural respondents in this study. It was seen as hindering LEOs from assisting livestock keepers, preventing zoonoses, and addressing meat safety risks. The Ministry of Livestock and Fisheries advocates a ratio of one LEO per village and recognises transport as a key ‘priority investment area’ for improving LEOs’ capacity [26, pers. comm. 7/11/2018]. One regional informant said the government tried to ensure all ward-level LEOs were issued motorbikes, although at the time of our interviews, only four of ten LEO respondents had them. The rest relied on their own motorbikes, hired transport, or walked. Access to a motorbike did not ensure effective performance however. Rural LEOs were often solely (or with only few colleagues) responsible for very large territories with poor roads that worsened during rainy seasons. Timeliness for slaughter inspection was a concern, and it was impossible to always be on site before slaughter occurred. Many LEOs had several distant sites to visit, most of which began operating before daybreak to ensure ‘warm’ meat was available for customers at the start of each day.

Shortage of material resources was another common concern for LEOs. In relation to inspection, uniforms and government ID cards were seen to provide LEOs and HOs with important symbolic authority. LEOs’ lack of uniforms was perceived as undercutting their ability to demand, as legislated, that butchers wear them - especially in urban areas. As one LEO put it, how could he insist on butchers wearing uniforms when he himself did not? Insufficient diagnostic tools also compromised LEOs’ field work. They perceived that when serious and/or unknown cases of animal disease were encountered, samples had to be sent for diagnosis to the district clinic in Moshi where they believed testing capacity was limited to anthrax or, more often, to the Veterinary Investigation Centre (VIC) in Arusha. This takes time and can lead to mistrust or at least, the danger that disease will spread in the meantime. And while trained in meat inspection techniques which make use of sight, smell, palpation, and incision (one claimed to ‘taste’ raw meat to detect medicine), they lacked technical equipment to support their inspections [53]. This meant that they could not immediately identify many zoonotic enteric pathogens, such as *Salmonella,* which do not manifest as illness in live animals or cause overt visual symptoms in meat, and yet may have significant negative consequences for human health. Indeed, such pathogens were not mentioned by LEOs or others, suggesting that they were not perceived to be significant threats. This is a well-recognised limitation of visual post-mortem meat inspection which, in many other contexts, has resulted in the emphasis and adoption of risk-based approaches.

Lack of clarity over, and farmers’ reluctance to pay for services historically provided freely by the government, represents another substantial challenge. While LEOs’ remit does not explicitly mandate (nor prohibit) the sale of drugs, treatment and other services, this practice was universally reported among our LEO respondents. They rationalised their sale of drugs and vaccines – which they purchased wholesale from private animal drug shops – as saving farmers from having to travel to town and paying cost prices, often for much larger doses than required for their animals. LEOs’ fees for drugs were mediated by prices in the private drug market, which varied geographically and over time, and by the distance and travel necessary to reach farmers. They claimed not to profit from these sales. However, many LEOs and other frontline actors perceived that farmers believed they were being exploited, and one LEO referred to ‘*other LEOs*’ who profit unfairly from drug sales. Variance in LEOs’ fees – based partly on costs and partly on LEOs’ assessments of what different clients could afford [25] – further exacerbated tension, particularly in relation to mandatory (yet not always free) vaccination campaigns. That *some* free vaccines were, although very rarely, provided by district-level authorities or donor projects in response to localised outbreaks of anthrax or rabies, created further confusion and spurred resistance. As one LEO explained:

*[…] last time we had rabies vaccines, we were asked to cover only villages bordering Kilimanjaro National Park [as these were considered most at risk]. We did as required, the rest paid. They complained but we showed them the letter from the District Council but they couldn’t understand. You know what that meant - others refused to vaccinate.* (Rural LEO, F)

Compounding many challenges highlighted above are LEOs’ and other frontline actors’ low pay. In addition to meagre baseline salaries, three LEOs with meat inspection duties mentioned they were entitled to extra pay, as their responsibilities required starting work as early as 3 am, and overtime on weekends and holidays when more animals are slaughtered. They had not received this extra pay for years, and consequently expressed demotivation and frustration. While there is evidence of Tanzanian frontline actors supplementing their low salaries through abuse of the system [59], no respondents admitted to such activities, although three non-LEO interviewees suspected bribery or ‘*collusion*’ occurring occasionally between meat inspectors and butchers and two LEOs mentioned refusing bribes.

Despite the many challenges and obvious frustrations, many LEOs and HOs remained motivated by recognition of the seriousness and necessity of their work in preventing disease and supporting livelihoods. In seeking to implement policy in a context characterised by transport and other material resource deficits, challenges related to drug and vaccination provision, low morale and inadequate pay, these frontline actors employ a range of strategies and skills to ensure meat safety.

### Strategies and skills for frontline action

Frontline actors’ main strategies to do their work included: using symbols of authority strategically; leveraging networks and teamwork; adapting to local contexts; and building local expertise. Each of these is discussed in turn below.

#### Symbols of authority: policy and officialdom

When asked about relevant legislation, most ward-level respondents made reference to ‘directives’ or local bylaws – which they explained provided them with guidance, legitimacy, and protection – rather than to national-level legislation. This corroborates previous government research that found little awareness among LEOs regarding specific laws such as the Animal Disease or Public Health Acts. Respondents indicated that ‘directives’ originated from ‘above’ – from authorities at ministry, regional or district level. In some cases, they may have been referring to regulations drawn up by ministries as directed in national Acts, while in others, they seemed to refer to ad-hoc measures or bylaws, the latter being developed at district, ward and village level [60]. Thus, lack of awareness around national laws – copies of which were not readily available to most – did not necessarily mean respondents were wholly unaware of their responsibilities and several spoke of learning about and discussing directives and bylaws at district or ward-level meetings, or receiving letters about them.

Enforcement situations – such as condemning and destroying unstamped meat, or threatening to close butcheries or eateries for failing to meet hygiene standards – were often tense, contested and difficult. It was therefore important for frontline actors to assert their authority. HOs, LEOs and committee volunteers would, as shown below, cite from or, if they had them, physically show livestock keepers and meat handlers relevant provisions in print.

*Before we destroy unsafe meat or food we have to read the sections of the bylaw covering food censure and destruction to the owner.* (Rural HO, F)

*Whenever we go to the site for inspection we normally take [the legislation] [ … ], we don’t scare business people. Before we take any action we educate and show them the section in the regulations [ … ]. You know many regulations are in English but we translate and explain some sections to the butchers and farmers.* (Urban LEO, M)

This strategy of appealing to officialdom – through taking along, reading aloud and explaining legislation – was usually referenced in relation to inspection of sites where meat was sold. It was seen, as indicated in the quote above, as embodying the necessary diplomacy and respect not to ‘*scare*’ business people while still encouraging their compliance. One LEO had requested an additional, official letter from the district council to reinforce his authority:

*We requested the district personnel to write the letter on our behalf to put more weight on it, as they respect and follow directives from higher levels more than from the ward.* (Rural LEO, M)

Resistance was sometimes met with threats of legal action, although few concrete examples were offered, in part perhaps, because the courts were not seen as very effective [61]. A more common strategy in such ‘*complicated cases’* was to request additional support from district or municipal-level authorities.

*They sometimes call us in to deal with someone who is not complying with the regulations, we go as a team to arrest the situation and make them comply.* (Urban District Livestock Officer, M)

*If they don’t respond and cooperate with me, recalling the previous incidents, then I will call for assistance from the municipal level.* (Urban HO, F)

While such appeals to ‘officialdom’ were usually made in the context of regulatory duties, some LEOs sought to similarly smooth tensions with farmers regarding treatment fees. They innovated mechanisms to ‘officialise’ service and drug fee charges. For one rural LEO, this took the form of letters from district authorities, and a system of official receipts:

*We asked the district council to write an official letter explaining to farmers they should pay for the services. The document will enable us to work smoothly with farmers. If an animal is suffering from say anaplasmosis, I will write a list of items to buy [ … ] and ask him or her to buy them from any veterinary shop. It’s always difficult for them to buy them.* *Then we negotiate the treatment price. Now we use special receipts from the district council.* (Rural LEO, F)

The need to regulate LEOs’ fee structures has also been recognised by government research as necessary to ‘make the system of livestock services more effective’ [25]. In the meantime, frontline actors make frequent appeals to ‘officialdom’ to legitimise their own authority and rely heavily on collaborative relationships.

#### Teamwork and leveraging networks

As illustrated above, meat condemnation can be difficult and contested as it involves destruction of property and income loss. Condemnation can happen at households where animals have died or been informally slaughtered; at slaughter sites (slabs or slaughterhouses); and at sites of meat sale. Although LEOs were sometimes invited by households or butchers to assess whether animals or carcasses were safe for consumption (in such instances, people were generally grateful, even if this resulted in condemnation), LEOs and HOs did at times discover or receive tip-offs from community members about suspicious animal deaths, slaughter, or meat sale. In such situations, and especially those in which human health was perceived to be clearly and/or immediately at risk, inspectors felt they had to act – but they often faced resistance or even, at times, personal danger. As such, frontline actors often drew on their professional networks to respond collectively:

*I had to form a small team of four, including the HO and two other meat inspectors from nearby wards. We arrived at the butchery, we didn’t ask many questions, we just condemned the meat.* (Urban LEO, F)

The above example involved a female LEO who subsequently worried the male butcher might ‘*hire people to harm’* her. Another rural female LEO’s diagnosis of anthrax was initially met with disbelief. ‘*Luckily*’, she explained, a retired male LEO and other staff had accompanied her, otherwise ‘*they would have harmed me or refused to bury* *[the carcass]*’. She recounted another incident of condemnation when a male livestock owner threatened her with a knife. She called a senior male colleague for backup. Such fears were not only experienced by female inspectors. One rural, experienced, male LEO worried about being poisoned, while another expressed safety concerns when commuting in the dark. Furthermore, during informal follow-up conversations between the author (TH) and two female officers (one LEO and one HO), these experiences were not perceived as particular to their gender. Such examples, nevertheless, demonstrate the importance of both men and women officers being able to draw upon a team for support when undertaking contentious acts of enforcement.

Frontline actors have, as indicated in the following quotes, become skilled at sensing when to call-in their colleagues:

*If I’m alone, it depends on the understanding of the butcher. If he accepts the truth we condemn it without problems but if he doesn’t, I call for assistance from other HOs from the municipal level and neighbouring wards to participate in the condemnation process.* (Urban HO, F)

*If I see an indication that the owner might bring problems later, I invite the District Vet Officer, HO and Solicitor, and fill in condemnation forms as required by law.* (Urban LEO, M)

Professional networks were not only important for difficult enforcement situations. Two LEOs reported using mobile phone apps to participate in informal LEO networks, seeking advice in the absence of regular training. Photographing a carcass and getting confirmation of a diagnosis bolsters LEOs’ confidence and can project authority while also informally sharing information about disease patterns:

*We have established an LEO group on smart phones. If one has a problem or needs clarification, we take photos and circulate them.* (Rural LEO, M)

*This network is very important as it provides opportunities to communicate to areas where [sick] animals are coming from [and tell them] to take control measures.* (Rural LEO, M)

Many frontline actors also drew upon local community networks and power structures for practical and political support. Announcements about vaccination campaigns or disease outbreaks were frequently made through religious organisations, savings and micro-finance groups, farmers groups, at markets and even funerals. LEOs and HOs – especially those in rural settings – also relied on local elected leaders at sub-village and street (urban) level to disseminate information, and to accompany them during vaccination campaigns or community hygiene inspections.^7^ In so doing, they relied on elected leaders’ good relationships with community members to encourage compliance.

*I will seek support from the chairpersons of the sub-villages. They are very powerful. People listen to them as they are elected by community members.* (Rural HO, F)

*We usually move around with village and sub-village leaders during official vaccination. They are very important as they participate in sensitization and people trust them.* (Rural LEO, M)

As indicated in these quotes, trust was central to accomplishing any work requiring the cooperation of residents, and local leaders were seen to be ‘*very close to their village members’*. They were capable of securing buy-in and participation beyond what officers could achieve given their inability to spend much time getting to know and delivering services in communities.

#### Adapting to local contexts

As frontline actors, LEOs and HOs were highly cognizant of the social and economic context in which they worked. They recognised that poverty affected people’s ability to pay for services, upgrade their premises, and comply with policy. Indeed, many respondents identified inability to afford services, drugs or upgrades as drivers of disease, and thus were sensitive to, and sought to accommodate, these local constraints. One rural LEO, quoted below, allowed poor livestock keepers to defer vaccination payments despite knowing reimbursement was unlikely. He and others also shared information about free vaccinations strategically:

*We don’t announce free vaccines. We announce the campaign, and then during the process and in collaboration with sub-village leaders, we identify weak families unable to pay and give them free vaccinations.* (Rural LEO, M)

Sensitivity to local conditions also helped LEOs and others recognise the difficulty of implementing certain regulatory recommendations – for instance that butchers have electric meat saws and deep freezers – leading them to overlook these when electricity was unavailable, unreliable, or unaffordable. Most focused instead on more context-appropriate regulations such as easily-cleanable tiled walls, glass windows, hand-washing facilities (running water hook-ups, or spigot-buckets) and plastic chopping boards, although the appropriateness of the latter was questioned by some.

LEOs’ and HOs’ sensitivity to poverty made them ‘*careful*’ and diplomatic in monitoring butcheries and meat handlers. One way of managing their relationships with butchers – which they were wary of damaging – was to selectively limit enforcement:

*Meat inspection is a sensitive job. I must be careful, otherwise I may damage good relationships with the butchers. After meat inspection, the rest of the work is done by the HO and other staff. I don’t want to follow business people that much, although I have to make sure the meat is safe to eat.* (Urban LEO, M)

This LEO emphasised making sure meat was inspected, choosing to leave ‘*the rest of the work’* – such as ensuring hygiene and infrastructural compliance – to others. During meat inspection at slaughter, several LEOs reported trying to minimise butchers’ losses:

*We feel very bad every time we discard animal livers. It’s a loss to butchers, but what can we do, we want consumers to eat safe meat. We may decide to trim the liver and remove affected areas to minimise loss*. (Rural LEO, M)

*When we find an animal with a disease that cannot affect other animals like dogs […], we call [the dog owners]. The owner of the cow would negotiate the price […] and can at least recover part of the loss incurred*. (Urban LEO, M)

LEOs used a number of strategies that included: rationalising non-enforcement of certain regulations, leaving work to HOs, and/or negotiating with butchers and farmers to upgrade their facilities and change their behaviour. While inspectors may have had personal ‘red lines’ in terms of minimum standards, HOs and LEOs alike emphasised a combination of flexibility and insistence:

*We explained to them the law requires all butcheries to meet hygiene standards. We had a tough time at first, but we sensitized them to the benefits and need for standards. We agreed on a deadline and all had to obey. I reminded them that if they didn’t make changes before the deadline we would not provide slaughtering services.* (Rural LEO, M)

*We have a lot to do, educating them to accept changes as a way of improving their business and safeguarding the health of their customers. We explain the possible consequences if slabs remains dirty allowing dogs and other animals scavenging on them. […] We educate them first, give them time to adopt the directed changes, if they don’t comply we finally use force.* (Rural HO, M)

LEOs and HOs understood the financial impact upgrades, business closures and fines could have, and that business people often claimed to be, or were unaware of regulations. Thus, they issued a series of reminders, warnings and deadlines before taking punitive action. One LEO with over two decades’ experience repeatedly emphasised the importance of what he called the ‘*extension approach*,’ the core of which, he explained, is communicating sensitively and diplomatically with butchers and meat handlers to gain their trust, educate them and encourage their compliance. This combination of flexibility, patience and skilful communication reflects recognition of both local socio-economic realities and social-cultural understandings of respectful interpersonal interaction in both urban and rural settings. For these reasons, LEOs and HOs did not simply mete out fines for non-compliance. Instead they explained ‘*the importance of implementing the law and the consequences of not complying’* (urban committee volunteer, F), confiscated unsafe meat, issued cautions and waited to see if improvements were implemented. When fines could no longer be delayed, frontline actors found ways to lessen the impact for those who could not afford to pay:

*This ward is one of the poorest in the Municipal Council. We understand nobody can pay that amount of money at once. They do it by instalment, or we may even forgive them.* (Urban HO, F)

In extreme cases of non-compliance, frontline actors did at times close businesses, but still they sought to limit associated financial burdens:

*After I’m satisfied with the work done, I allow them to continue business again. We don’t ask them to pay a fine, because that would be double punishment.* (Rural committee volunteer, F)

What is clear in the above examples is frontline actors’ use of discretion and diplomacy to carry out their duties in ways sensitive to local economic realities, social norms and expectations. This allows them to negotiate behaviour change in ways that preserve, in as far as possible given the nature of their work, relationships of trust with butchers and others.

#### Building local expertise and capacity

Our respondents emphasised the importance of education and awareness-raising about animal health, meat safety, and human disease. This ranged from advising consumers about safe meat consumption, to teaching farmers and butchers to recognise signs of animal illness and unsafe meat and explaining why certain protocols and standards existed. Such instruction happens through campaigns, meetings, training, media, and religious forums, and involve both independent and collaborative efforts. Clinical personnel for instance, described disease prevention efforts through in-clinic training.

*Health education is done every day at the dispensary. It’s like prayers, we have fixed timetables showing that today we have health education on nutrition, on malaria,* etc. *If there is an outbreak of animal disease, we also include it in the sessions.* (Urban nurse, F)

Community volunteers serving on ward and village or street-level health and environment committees, often in collaboration with HOs, taught local residents to protect themselves and others by buying only inspected meat, and encouraged its thorough cooking.

*[At public gatherings] we tell them not to eat un-inspected meat and the government stamp on the meat means inspection was done and it is safe to eat.* (Rural committee volunteer, F)

*I talk a lot with mama lishe [food sellers] on proper ways of food preparation […] they have to make sure that food is cooked for a long-time in a clean environment.* (Urban HO, F)

Certain frontline actors recognised the importance of ‘meeting’ people where they are. They therefore stressed their efforts, despite resource constraints, to visit far-flung corners of a ward, recognising they might, through education rather than timely inspection, prevent deaths from consuming infected meat. They also recognised that many people did not have capacity or inclination to attend public functions or meetings. In the words of one rural LEO referring to past government efforts to convene farmer groups for training purposes:

*They have other issues to deal with, they cannot waste time listening to facilitators for several hours without getting anything tangible at the end of the day. ‘What shall we eat this evening, your words? We have families we need to feed, we cannot waste our time listening to you.’* (Rural LEO, M)

LEOs therefore also built training into their individual household visits.

*We educate family members every time we visit for animal treatment. We tell them the symptoms of animal diseases, how they are transmitted and how to control them.* (Rural LEO, M)

Indeed, LEOs’ mandated duties to support animal-based livelihoods include such instruction. However, they broadened the remit of their prescribed educational duties to include coaching butchers to identify unsafe meat and understand why particular standards were necessary.

*We educate them on the consequences of butcheries without required infrastructure. [We explain that] meat which comes in contact with flies may harm their consumers. We demonstrate the difference between a wall covered with tiles and one not. If the blood splashes on a wall without tiles, how would they clean it?* (Urban LEO, F)

This strategy – of training butchers so they might self-regulate – helped LEOs and others address their own limited capacity to serve and inspect all places punctually.

*It may happen I am late to the slaughter site, I allow them to continue selling meat if no unusual symptoms have been seen on the animal carcass. I’m glad no one has ever betrayed my trust. They know how to examine the meat, I always show them ….* (Rural LEO, M)

One LEO also linked butchers’ understandings of meat safety to the mitigation of conflict should he have to condemn meat:

*So we have to educate [butchers] why the meat must be thrown away. Some understand. There must be obvious reasons and fortunately they can see this with their own eyes. So we do a lot of counselling and they sometimes do their own observation before I arrive. They know beforehand that today there will be no lungs or kidneys or heart* etc. *When I arrive they keep quiet to hear the final decision from me.* (Rural LEO, M)

Education was thus, not only a way of ‘*sensitising*’ community members to ‘*the benefits of complying with the laws and regulations on health issues*’ (urban HO, M) and of raising general consciousness about human and animal health, it was also about being fair in a resource-strapped context. As shown above, punitive action for non-compliance was often treated as a last resort, meted out only after considerable effort to inform people of rules, standards and associated rationales.

## Discussion

The concept ‘street-level diplomacy’ makes visible and better theorises the skills which enable frontline actors to implement policy, despite limited material resources, and institutional and cultural barriers [37]. It brings the bureaucratic dimensions of rules, procedures, and decision-making together with more diplomatic aspects of negotiation, communication and persuasion and draws attention to frontline actors’ ‘soft’ power to create and leverage networks, and to engage, build trust and persuade individuals to comply with policy. In contrast to much previous work which emphasises street-level bureaucrats’ undermining power, Gale and colleagues call attention to the power of frontline actors’ discretionary use of *diplomacy* to work towards policy goals through seemingly unorthodox routes.

The data described above reinforces the notion that frontline actors do not always implement regulations and policy as envisioned by high-level policy makers. Rather, exercising discretion and autonomy in their day-to-day activities, they adapt to contexts in which they work, innovating on legislative prescriptions, leveraging informal strategies, and choosing not to enforce at times. Literature on street-level bureaucrats often frames such behaviour as necessary (particularly in the light of scarce resources), yet resulting in ‘selective implementation’ or problematic divergence from high-level policy aims [29, 30] and, at times, this may indeed be the case. Like Gale and colleagues [37] however, this paper offers an alternative perspective highlighting how frontline actors use their discretion and a range of diplomatic skills and strategies to approximate higher policy goals. Indeed, in low-resource contexts, such skills and strategies have been described by Funder and Marani as ‘a prerequisite of the African state’. They note that environmental officers in Kenya occupy ‘an ambiguous position in which they are expected to implement lofty laws and policies with limited means and in a complex local reality’ and that their ability to navigate this creatively was essential to ‘keep this old truck on the road’ [62].

To accomplish this in the Tanzanian context, the frontline actors we interviewed used a blend of formal and informal strategies and soft and hard power to tailor their mandates to complex realities and individual encounters. This allowed them to function creatively despite constraints, and more specifically, to build trust with communities they worked in, boost their own capacity and legitimacy, and develop local residents’ ability to self-regulate.

### Building and maintaining trust

Trust, while recognised in literature on street-level bureaucrats [30], is an underappreciated dimension in local-level regulatory and service provision activities [63]. If people mistrust frontline actors, they are likely to be suspicious of the behaviours and practices frontline actors would like them to adopt, or even of the services they provide [38]. Mugambi and colleagues found in Kenya for instance, that pastoralists remained suspicious of biomedicine and LEOs’ motives due to infrequent encounters and negative experiences [64]. Similar histories of distrust taint Tanzanian extension workers [65, 66]. While LEOs, HOs and others in our study did not explicitly claim they worked to build trust among people to approximate policy goals, they nevertheless articulated strategies that do this.

Cognizant that people were not necessarily aware of regulations, and that many were poor, frontline actors stressed the importance of carefully explaining and counselling to butchers exactly what the rules and standards were, and why they should be followed, or in the case of livestock keepers, why they should participate in vaccination campaigns and call LEOs in the event of animal death or illness. Non-compliance, especially if interpreted to result from a lack of awareness or inability to afford necessary changes, was met with patience and flexibility and ‘*double punishment*’ – fines on top of business closure or meat condemnation –avoided. These strategies reflect frontline actors’ sensitivity to local economic capacity and recognition of the importance of fairness. They knew, not only that it was unrealistic to expect immediate investments and compliance, but that they also needed to build and maintain positive relationships: ‘*I understand we have to enforce laws, but sometimes you have to act with caution to avoid conflicts and misunderstandings*’ (urban HO, F). In this way, choosing at times *not* to enforce was a strategy in and of itself. This was best illustrated by LEOs who inspected meat but overlooked hygiene issues so as not to create unreasonable difficulties for businesses or, as one phrased it, to not *‘follow business people that much’* (urban LEO, M). In this way, they could avoid the risk of being seen as too demanding, preserve space for positive relations, and thus potentially make future cooperation more likely. Dickinson and Sullivan [67] see policy implementers’ attempts to engage with local people’s values as a form of cultural performance which, through ‘contextualized interaction’, builds trust. They argue that such behaviours enhance policy efficacy and implementation because, in engaging with local social norms, frontline actors are also affirming values, constituting meaning and building social efficacy.

Frontline actors’ authority and trustworthiness was also boosted by working collaboratively with elected leaders, especially during animal vaccination campaigns and hygiene inspections, and through engagement with community institutions, like churches and mosques, to announce campaigns or disease outbreaks. Such collaboration aligned disease control and meat safety with locally-recognised and respected networks which people freely associated with, and with locally elected leadership. While this may not necessarily guarantee widespread awareness or ensure vaccination coverage [38], it illustrates LEOs’ and HOs’ recognition of the importance of networks of trust for policy implementation. Carey et al. argue, that trust is ‘critical to implementation’ in complex, dynamic and highly-relational contemporary contexts [28]. It enhances cooperation, reduces transaction costs, increases predictability and reduces opportunistic behaviour [63]. In keeping with this, LEOs’, HOs’ and other’s accounts show they have nurtured trust to enhance regulation and implementation of meat safety in northern Tanzania.

### Boosting frontline actors’ regulatory capacity

Despite frontline actors’ patience, flexibility and diplomacy, there were times they felt they had to act, such as when human health was immediately at risk. This included having to condemn unsafe or uninspected meat, or dealing with individuals’ or businesses’ repeated or gross non-compliance. When such instances occurred, they relied on formality – on professional relationships, hierarchy, and policy documents – to boost their capacity for regulation and enforcement. These can be seen as symbols which enhance the efficacy of the regulatory process [28]. Although meat and animal condemnation technically required the presence of multiple frontline actors and, in some instances, an official laboratory diagnosis, LEOs and HOs described scenarios in which they, as individuals, were sufficiently confident to condemn and destroy meat.^8^ In situations of active resistance, LEOs and others called upon colleagues, including district-level superiors, to back them up. This served to allocate responsibility for punitive measures – which did on occasion cause conflict and fear – higher up in government or across a team. In so doing, these frontline actors demonstrated their ability to ‘draw different policy strands together, to reconcile competing priorities’, and ‘to relinquish control while managing risk’ [28] while also pronouncing defined policy goals.

Indeed, contrary to much literature on street-level bureaucrats, which sees frontline actors as ‘shirking or sabotaging’ official responsibilities [30], these LEOs and HOs regarded formal policy positively [36]. They saw it not only as providing clear and necessary guidelines to safeguard public health, but as bestowing on them legitimacy and authority to act, and granting them legal protection. Some frontline actors had copies of policy texts or special letters from district authorities which they carried and read aloud *‘to put more weight’* on what they asked of people. This tactic was used in delicate situations of immediate concern (such as confiscating meat), and as part of a range of diplomatic strategies to persuade people to engage in long-term infrastructural, procedural or behavioural change. But regulation and authority were not, on their own, enough. As demonstrated above, LEOs and HOs emphasised the need to explain regulations carefully to ensure people recognised they were not being treated unfairly, and that there was good reasoning behind these stipulations.

### Promoting self-regulation

Education and advice is central to extension work and is aimed at empowering farmers and livestock keepers to optimise their livelihoods, yet frontline actors also saw this as a means of redistributing responsibility, and hedging risk. Constrained by material and institutional challenges, frontline actors were under-resourced and unable to be everywhere they needed to be. They worried that disease might erupt with disastrous health consequences, and that they would be blamed for this. A central theme of Lipsky’s theory on street-level bureaucrats is that these actors seek maximum information asymmetry between themselves, and the citizens they work with to prevent their decisions being questioned [30, 31]. In Tanzania, however, LEOs and HOs pursued a different strategy. LEOs with meat inspection duties emphasized their training of farmers and butchers to recognise visible signs of disease not merely to fulfil their duties as extension workers, but to maximise meat safety in their inevitable absences, especially in rural areas. Because they were incapable of overseeing all butchers’ activities, and yet were concerned that all meat was safe, some rural LEOs developed informal agreements with butchers. They spent time teaching butchers to identify visibly unsafe meat with the understanding that when they were late or absent, as they inevitably would be at times, the butchers would be responsible and not to sell it.

*During rainy season, all roads are impassable […] It is not easy to visit […] some of the slaughter sites. That is why I decided to train meat attendants and butchers in basic knowledge in identifying infectious animal disease and to inform me immediately.* (Rural LEO, M)

In proposing the concept of ‘street-level diplomacy’, Gale and colleagues argue that ‘diplomatic activity does not seek to enforce or coerce, but to facilitate through assisting partners to take responsibility for their own changes’ [37]. By rejecting the language of coercion and enforcement and by adopting the more cooperative language of ‘training’, ‘assisting’, and ‘informing’, the emphasis shifts from LEOs’ punitive and patronising imposition of policy to a relationship that involves coaching for empowerment, capacity building among food safety actors and space for collaboration [68, 69]. For frontline actors working for animal health and meat safety in resource-constrained contexts, this also helps generate the trust and buy-in needed when, as health threats emerge, sensitive, decisive, and unpopular decisions have to be taken – while easing some of the LEOs’ burden of responsibility for disease prevention.

Funder and Marani, who identified similar frontline actor-civil society relationships in the management of Kenya’s sensitive ecosystems, argued that ‘the risk of “sharing” this authority’ was ‘far outweighed by the legitimacy’ it provided [62]. By putting so much emphasis on education, and communicating trust that butchers and farmers would do the right thing in policy implementers’ absence, LEOs and HOs still face the possibility that people will not comply. Yet, by meeting this risk – which is ultimately impossible to avoid – with an inclusive, diplomatic approach, they attempt to minimise the potential for disease and illness. As Mackintosh and Tibandebage argue in relation to Tanzania’s drug inspectors, ‘Effective regulatory intervention is only possible in Tanzania if the resource constraint on inspection and enforcement can largely be side-stepped’ [61]. An approach which engages livestock owners and meat sellers as regulatory partners offers a means of achieving this for meat safety.

This study focused upon the practices and understandings of the inspectors. For a more complete picture of the inspection process, butchers’ and slaughter workers’ views and practices should also be considered. This is an important component of red meat safety in Tanzania which deserves academic attention. Moreover, while our results do not suggest significant differences in the practices, understandings and experiences of male and female staff, an important future study which further explores patriarchal dimensions of this work more clearly is merited. These limitations do not detract from this study’s valuable insights into how Tanzania’s frontline actors use street-level diplomacy in their attempts to ensure meat safety.

## Conclusion

Frontline actors charged with ensuring meat safety in northern Tanzania face a tension between asserting authority and making demands, and building trusting relationships with those they are mandated to regulate and serve. As shown in this paper, they navigate this tension by drawing on a range of strategies and techniques, both formal and informal, tailored to specific situations and contexts. Mediated by their limited capacity and inability to be everywhere they need to be, they stress the importance of maintaining positive relationships, and do so by deploying ‘street-level diplomacy’. This included sensitivity to local socio-economic realities, and respecting local social norms and expectations around politeness, fairness and reciprocity. They communicated carefully with livestock owners and meat sellers, recruited the support of influential community leaders and institutions, explained the importance of regulations, provided grace periods to rectify non-emergency non-compliance, and even dismissed minor infractions, especially when they could not fulfil their own duties. They hesitated to strictly enforce regulations, and sought to minimize financial loss stemming from regulatory activities. In choosing to prioritise only some issues, and not to interfere too much in butchers’ livelihoods, they preserved positive relations.

Education and awareness were seen by these frontline actors as essential to livelihood activities and to mitigate meat safety risks. Underpinning this was a belief that sensitising people is key to creating change. What these frontline actors understood, however, was that a generic approach with education at its core was not enough. Rather, livestock owners and meat sellers had to be made to feel respected, that they were being treated fairly, and that they were trusted to make good decisions. This encouraged them to learn from inspectors, and to comply with ‘reasonable’ regulations, while knowing inspectors would not impose livelihood damaging expectations and standards. It also made it easier for frontline actors to take difficult, yet occasionally necessary actions (condemn animals or meat, or shut down businesses) which may thus be perceived more fairly, elicit less resistance, and encourage cooperation. Recognising this diplomacy allows for seeing ‘non-enforcement’ in a new light, at least in certain circumstances: as not just the result of LEOs’ limited capacity, but as a deliberate, if unorthodox strategy to ensure meat safety.

By highlighting these strategies and skills, we do not intend to romanticise the capabilities or intentions of frontline actors, or even the downstream consequences of their actions. It remains likely that some are engaged in exploitative or clientalist practices, especially regarding service provision. Moreover, these diplomatic skills and strategies are not always sufficient for ensuring meat safety as, for example, they cannot influence the affordability of infrastructural improvements. We witnessed little attention to hazards such as enteric pathogens which, given their invisibility, may make frontline actors more likely to pass them over when making decisions about what standards to enforce. Yet, in resource-constrained contexts, street-level diplomacy may represent the ‘best possible way to get things done under the circumstances’ [62]; and in the absence of adequate staff, frontline actors’ flexibility around enforcement may make them unique ‘drivers of policy’. These seemingly inefficient, yet ‘*de facto* bureaucratic policy makers’ may contribute to more appropriate policy implementation while simultaneously ‘promoting local democratic control and tailoring policies to local needs’ [61]. Thus, when considering ways to strengthen meat safety policies in contexts such as that discussed here, rather than seeing the partial enforcement of regulations as a failure, it may be more productive to consider ways of working with frontline actors to enhance what they and others can reasonably do under the constraints they currently face.

We propose that the knowledge produced in this study suggests (in addition to increasing resource availability) that LEOs and other frontline actors be more included in policy making processes, and be provided with opportunities to frankly discuss how social, economic and cultural realities mediate their work, and share strategies for maximising their capacities. This could also recognise the role of training and networking opportunities which go beyond the technical aspects of their work to consider more explicitly the role of trust, and the delicate navigation of relationships.

## Additional files

Additional file 1: Interview schedule for administrative staff, elected officials and community health volunteers. (DOCX 20 kb)

Additional file 2: Interview schedule for frontline technical staff (LEOs, HOs, clinical personnel) (DOCX 21 kb)

## Data Availability

The datasets used and/or analysed during the current study are currently available from the corresponding author on reasonable request, and will be lodged in a public repository at the culmination of the project.

## Abbreviations

HO: Health Officer
LEO: Livestock Extension Officer
LFO: Livestock Field Officer
LMIC: low- and middle-income country
MERS: Middle East respiratory syndrome
RVF: Rift Valley fever
SARS: Severe acute respiratory syndrome
VIC: Veterinary Investigation Centre

## Footnotes

1 LEOs, officially known as Livestock Field Officers (LFOs), typically receive a diploma in animal health or production at a specialised livestock training institute [25]. HOs are trained in the monitoring and management of disease outbreaks and communicable diseases

2 In some cases – such as with LEOs and community volunteers – there was more than one individual holding a particular position. The decision who to interview was made by the ward-level head of the relevant department. For instance, a lead LEO appointed a colleague, while HOs appointed particular community volunteers from their wards.

3 The majority of red meat butchers in Tanzania, and much of East Africa, are male. This stems from long-standing cultural traditions stipulating that men are the owners, managers and handlers of cattle.

4 Two interview schedules were used, covering the same topics but tailored to the respondents’ roles (either technical or administrative/elected). See Additional file 1 and Additional file 2

5 Slaughterhouses are enclosed or roofed buildings, and are generally larger than slabs. They tend to operate in more systematic and formal ways, and have more developed infrastructure (drainage systems and running water) and equipment (such as hooks). Slaughterhouses are not, however, as highly formal or mechanized as abattoirs, such as the main facility in Arusha [53].

6 Although not officially required, Regional and District Commissioners were reported to have insisted on glass windows because of the visibility offered and ease of cleaning.

7 During community hygiene inspections, HOs scrutinise neighbourhoods, examining sanitation and the conditions animals are kept in.

8 Unsafe meat or animal carcasses were buried with lime and kerosene in serious cases. Sometimes, such as in anthrax cases, the buried carcass was fenced off to discourage animals from unearthing it.

## References

[1] Wood J. L. N., Leach M., Waldman L., MacGregor H., Fooks A. R., Jones K. E., Restif O., Dechmann D., Hayman D. T. S., Baker K. S., Peel A. J., Kamins A. O., Fahr J., Ntiamoa-Baidu Y., Suu-Ire R., Breiman R. F., Epstein J. H., Field H. E., Cunningham A. A. (2012). A framework for the study of zoonotic disease emergence and its drivers: spillover of bat pathogens as a case study. Philosophical Transactions of the Royal Society B: Biological Sciences.

[2] Kilpatrick AM, Randolph SE (2012). Drivers, dynamics, and control of emerging vector-borne zoonotic diseases. Lancet.

[3] Broglia A, Kapel C (2011). Changing dietary habits in a changing world: emerging drivers for the transmission of foodborne parasitic zoonoses. Vet Parasitol.

[4] Dry S, Leach M, editors. Epidemics: science, governance and social justice. London: Earthscan; 2010.

[5] Morens DM, Folkers GK, Fauci AS (2004). The challenge of emerging and re-emerging infectious diseases. Nature.

[6] Jones BA, Grace D, Kock R, Alonso S, Rushton J, Said MY (2013). Zoonosis emergence linked to agricultural intensification and environmental change. PNAS..

[7] Grace D, Mutua F, Ochungo P, Kruska R, Jones K, Brierley L (2012). Mapping of Poverty and Likely Zoonoses Hotspots.

[8] Karimuribo ED, Mboera LEG, Mbugi E, Simba A, Kivaria FM, Mmbuji P (2011). Are we prepared for emerging and re-emerging diseases? Experience and lessons from epidemics that occurred in Tanzania during the last five decades. Tanzan J Health Res.

[9] Allen T, Murray KA, Zambrana-Torrelio C, Morse SS, Rondinini C, Di Marco M (2017). Global hotspots and correlates of emerging zoonotic diseases. Nat Commun.

[10] Sindato C, Karimuribo E, Mboera LE. The epidemiology and socio-economic impact of Rift Valley fever in Tanzania: a review. Tanzan J Health Res. 2011;13(5):1-16. 10.4314/thrb.v13i5.1 26591986

[11] Peyre M, Chevalier V, Abdo-Salem S, Velthuis A, Antoine-Moussiaux N, Thiry E (2015). A systematic scoping study of the socio-economic impact of Rift Valley fever: research gaps and needs. Zoonoses Public Health.

[12] Allan Kathryn J., Biggs Holly M., Halliday Jo E. B., Kazwala Rudovick R., Maro Venance P., Cleaveland Sarah, Crump John A. (2015). Epidemiology of Leptospirosis in Africa: A Systematic Review of a Neglected Zoonosis and a Paradigm for ‘One Health’ in Africa. PLOS Neglected Tropical Diseases.

[13] Crump John A., Morrissey Anne B., Nicholson William L., Massung Robert F., Stoddard Robyn A., Galloway Renee L., Ooi Eng Eong, Maro Venance P., Saganda Wilbrod, Kinabo Grace D., Muiruri Charles, Bartlett John A. (2013). Etiology of Severe Non-malaria Febrile Illness in Northern Tanzania: A Prospective Cohort Study. PLoS Neglected Tropical Diseases.

[14] Reddy Elizabeth A, Shaw Andrea V, Crump John A (2010). Community-acquired bloodstream infections in Africa: a systematic review and meta-analysis. The Lancet Infectious Diseases.

[15] Halliday J, Daborn C, Auty H, Mtema Z, Lembo T, deC Bronsvoort BM (2012). Bringing together emerging and endemic zoonoses surveillance: shared challenges and a common solution. Philos Trans Biol Sci.

[16] Swai ES, Schoonman L, Daborn CJ (2010). Knowledge and attitude towards zoonoses among animal health workers and livestock keepers in Arusha and Tanga. Tanzania Tanzan J Health Res.

[17] Dlamini Beauty Sicelo, Montso Peter Kotsoana, Kumar Ajay, Ateba Collins Njie (2018). Distribution of virulence factors, determinants of antibiotic resistance and molecular fingerprinting of Salmonella species isolated from cattle and beef samples: suggestive evidence of animal-to-meat contamination. Environmental Science and Pollution Research.

[18] MLFD. 2015. Tanzania livestock modernization initiative. Ministry of Livestock and Fisheries Development, July 2015.

[19] Wilson RT. 2015 The red meat value chain in Tanzania: a report from the southern highlands food systems programme.

[20] Mbwambo MSN, Mruttu H, Dotto M, Ndomba C, da Silva M, Makusaro F (2018). Tanzania livestock master plan.

[21] UNIDO (2012). Tanzania’s red meat value chain: a diagnostic, Africa agribusiness and agroindustry development initiative (3ADI) reports.

[22] Alphonce R, Alfnes F (2012). Consumer willingness to pay for food safety in Tanzania: an incentive-aligned conjoint analysis. Int J Consum Stud.

[23] Kurwijila L, Mwingira J, Karimuribo E, Shirima G, Lema B, Ryoba R (2011). Safety of animal source foods in Tanzania: a situational analysis.

[24] United Republic of Tanzania, Prime Minister’s Office, n.d., One Health Strategic Plan: 2015–2020; WHO, 2018. Tanzania commits to embrace the One Health approach, WHO Regional Office for Africa, https://afro.who.int/news/tanzania-commits-embrace-one-health-approach. Accessed 16 Nov 2018.

[25] MALF. 2016. Livestock field officer survey: policy priorities for improved livestock services. Ministry of Agriculture, Livestock and Fisheries.

[26] Bech MM, Lawi YQ, Massay DA, Rekdal OB (2013). Changing policies and their influence on government health workers in Tanzania, 1967–2009: perspectives from rural Mbulu district. Int J Afr Hist Stud.

[27] Nilsen P, Ståhl C, Roback K, Cairney P (2013). Never the twain shall meet? A comparison of implementation science and policy implementation research. Implement Sci.

[28] Carey Gemma, Dickinson Helen, Olney Sue (2019). What can feminist theory offer policy implementation challenges?. Evidence & Policy: A Journal of Research, Debate and Practice.

[29] Lipsky M (1980). Street-level bureaucracy: dilemmas of the individual in public services.

[30] Sevä Mikael, Jagers Sverker C. (2013). Inspecting environmental management from within: The role of street-level bureaucrats in environmental policy implementation. Journal of Environmental Management.

[31] Arnold Gwen (2014). Policy learning and science policy innovation adoption by street-level bureaucrats. Journal of Public Policy.

[32] Hill M, Hupe P (2009). Implementing public policy.

[33] Long N (2001). Development sociology: actor perspectives. London.

[34] Degnbol T, Benjaminsen TA, Lund C (2001). Inside government extension agencies: a comparison of four agencies in the Sikasso region of Mali. Politics, Property and Production in the West African Sahel: Understanding Natural Resource Management.

[35] Cook EAJ, de Glanville WA, Thomas LF, Kariuki S, de Clare Bronsvoort BM, Fèvre EM (2017). Working conditions and public health risks in slaughterhouses in western Kenya. BMC Public Health.

[36] Tummers LG, Bekkers VJJM (2014). Policy implementation, street-level bureaucracy and the importance of discretion. Public Manag Rev.

[37] Gale Nicola, Dowswell George, Greenfield Sheila, Marshall Tom (2017). Street-level diplomacy? Communicative and adaptive work at the front line of implementing public health policies in primary care. Social Science & Medicine.

[38] Bardosh K (2014). Global aspirations, local realities: the role of social science research in controlling neglected tropical diseases. Infect Dis Poverty.

[39] Nørrung B, Buncic S (2008). Microbial safety of meat in the European Union. Meat Sci.

[40] Ladbury G, Allan KJ, Cleaveland S, Davis A, de Glanville WA, Forde TL (2017). One health research in northern Tanzania – challenges and progress. East African Health Res J.

[41] Grace D (2015). Food safety in low and middle income countries. Int J Environ Res Public Health.

[42] Shun-King M, Gilson L, Mbachu C, Molyneux S, Muraya KW, Uguru N, Govender V (2018). Leadership experiences and practices of south African health managers: what is the influence of gender? -a qualitative exploratory study. Int J Health Equity.

[43] Adjiwanou V, LeGrand T (2014). Gender inequality and the use of maternal healthcare services in rural sub-Saharan Africa. Health Place.

[44] Oliver M, Geniets A, Winters N, Rega I, Mbae SM (2015). What do community health workers have to say about their work, and how can this inform improved programme design? A case study with CHWs within Kenya. Glob Health Action.

[45] Temple B, Young A (2004). Qualitative research and translation dilemmas. Qualitative research.

[46] Hsieh Hsiu-Fang, Shannon Sarah E. (2005). Three Approaches to Qualitative Content Analysis. Qualitative Health Research.

[47] Saldaña J (2016). The coding manual for qualitative Reseachers.

[48] Schreier M (2012). Qualitative content analysis in practice.

[49] Rutabanzibwa AP. Veterinary legal reform in Tanzania. In: Proceedings of Primary Animal Health Care in the 21st Century: Shaping the Rules, Policies and Institutions, Mombasa, Kenya; 2002. http://agris.fao.org/agris-search/search.do?recordID=GB2013203851. Accessed 5 June 2019.

[50] Swai ES, Masaaza S, Daborn CJ (2014). Evaluation of community animal delivery systems in Simanjiro, Tanzania. Livest Res Rural Dev.

[51] Mwakapeje E, Høgset S, Fyumagwa R, Nonga HE, Mdegela R, Skjerve E. Anthrax outbreaks in the humans-livestock and wildlife interface areas of northern Tanzania: a retrospective record review 2006-2016. BMC Public Health. 2018;18. 10.1186/s12889-017-5007-z PMC5755297 29304765

[52] Jabbar MA, Grace D (2012). Regulations for safety of animal source foods in selected Sub-Saharan African countries: Current status and their implications.

[53] United Republic of Tanzania, 2016. A Performance Audit Report on the Hygiene Control in Meat Production.

[54] Komba EV, Mkupasi EM, Mbyuzi AO, Mshamu S, Mzula A, Luwumba D. Sanitary practices and occurrence of zoonotic conditions in cattle at slaughter in Morogoro municipality, Tanzania: implications for public health. Tanzan J Health Res. 2012;14(2):1-12. 10.4314/thrb.v14i2.6 26591734

[55] Kamani TM, Kazwala R, Mfinanga S (2015). One health: a concept led by Africa, with global benefits. Vet Rec.

[56] CDC. One health zoonotic disease prioritization for multisectoral engagement in Tanzania. Workshop Summary. Dar es Salaam: March 23-24th 2017, https://www.cdc.gov/onehealth/pdfs/tanzania-report-508.pdf . Accessed 13 Nov 2018.

[57] Caudell MA, Quinlan MB, Subbiah M, Call DR, Roulette CJ, Roulette JW (2017). Antimicrobial use and veterinary care among agro-pastoralists in northern Tanzania. PLoS One.

[58] Gustafson CR, VanWormer E, Kazwala R, Makweta A, Paul G, Smith W (2015). Educating pastoralists and extension officers on diverse livestock diseases in a changing environment in Tanzania. Pastoralism..

[59] Cooksey B (2012). Politics, patronage, and projects: the political economy of agricultural policy in Tanzania. Future Agric Consortium Working Paper.

[60] Mowo J, Masuki K, Lyamchai C, Tanui J, Adimassu Z, Kamugisha R (2016). By-laws formulation and enforcement in natural resource management: lessons from the highlands of eastern Africa. Forests, Trees Livelihoods.

[61] Goodman C., Kachur S P., Abdulla S., Bloland P., Mills A. (2007). Drug shop regulation and malaria treatment in Tanzania why do shops break the rules, and does it matter?. Health Policy and Planning.

[62] Funder M, Marani M (2015). Local bureaucrats as bricoleurs. The everyday implementation practices of county environment officers in rural Kenya. Int J Commons.

[63] Cerna L. Trust: what it is and why it matters for governance and education. OECD Education Working Papers. 2014;108:5-66.

[64] Mugambi JM, Wesonga FD, Ndungu SG (2012). Ticks and tick-borne disease control in a pastoral and an agro-pastoral farming system in Kenya. Livest Res Rural Dev.

[65] Sunseri T (2013). A political ecology of beef in colonial Tanzania and the global periphery, 1864–1961. J Hist Geogr.

[66] Rwongezibwa BM (2009). Provision of livestock extension services in per-urban areas: A case of Kitunda Ward, Ilala Municipality, Dar-Es-Salaam Region. Masters Dissertation.

[67] Dickinson H, Sullivan H (2014). Towards a general theory of collaborative performance. Public Adm.

[68] Nguyen-Viet H, Tuyet-Hanh TT, Unger F, Dang-Xuan S, Grace D (2017). Food safety in Vietnam: where we are at and what we can learn from international experiences. Infect Dis Poverty..

[69] Chotinun S, Rojanasthien S, Unger F, Suwan M, Tadee P, Patchanee P (2014). An integrative approach to enhancing small-scale poultry slaughterhouses by addressing regulations and food safety in northern-Thailand. Infect Dis Poverty.

