# Supplementary material for: Street-level diplomacy and local enforcement for meat safety in northern Tanzania: knowledge, pragmatism and trust
Source: BMC Public Health. 2019 Jul 3;19:863. doi: 10.1186/s12889-019-7067-8 (PMC6610827; doi:10.1186/s12889-019-7067-8)
Supplement: Supplementary file 1 — Interview schedule for administrative staff, elected officials and community health volunteers. (DOCX 20 kb) [file 12889_2019_7067_MOESM1_ESM.docx]

APPENDIX A

# Interview Schedule 1

Interview schedule for administrative staff, elected officials and community health volunteers

## A. Work and duties

1. Can you tell me about your work as [job title]? What do you do?
2. Do you think the practices around slaughter and sale of meat have changed over the past 5-10 years, how? What do you think has caused these changes?
3. How would you as a [job title] become informed of a disease being transmitted from animals/meat to humans that you have to deal with?

## B. Challenges in securing food safety of meat

1. In the past 5-10 years, what new policies have been introduced and which policies have improved?
2. If there were reports of a risk or actual occurrence of diseases being transmitted from animals to humans, what would you do? What is your specific role?
3. What difficulties do you face in making sure people are protected from animal diseases?
4. In your job as [job title], have you had any successes at identifying or preventing animal-to-human disease? What were these?

## C. Policies and regulations helping/hindering

1. What kinds of places/processes do you inspect or are you responsible for (in relation to animal-human disease)? Who do you report to?
2. Do you have specific legislation/policies that you have to enforce? What are these?
3. In your work, are there specific policies that help you, or make it difficult for you, to ensure that diseases don’t pass from animals to humans? Can you explain more?
4. How has decentralisation-by-devolution affected your work?

## D. Shocks re food safety of meat

1. In all the years that you have been involved in this work, have you ever had any major events or problems with diseases? What were these? How did this affect your role and relationships?
2. Do you think there will be any major problems regarding diseases passing from animals to humans in the future? What will these be and what will cause them?
3. Do you think there is more that can be done to control animal diseases and keep people safe? Who should take responsibility for this?
4. What do you think of the Abattoir in Arusha as a means to slaughter animals and control disease?
5. Would you like to make any other comments?
