# Supplementary material for: Street-level diplomacy and local enforcement for meat safety in northern Tanzania: knowledge, pragmatism and trust
Source: BMC Public Health. 2019 Jul 3;19:863. doi: 10.1186/s12889-019-7067-8 (PMC6610827; doi:10.1186/s12889-019-7067-8)
Supplement: Supplementary file 2 — Interview schedule for frontline technical staff (LEOs, HOs, clinical personnel) (DOCX 21 kb) [file 12889_2019_7067_MOESM2_ESM.docx]

APPENDIX B

# Interview Schedule 2

Interview schedule for frontline technical staff (LEOs, HOs, clinical personnel)

## A. Work and duties

1. Can you tell me about your work as [job title]? What do you do?
2. Does your work involve butchers and meat sellers? How?
3. Do you think the practices around slaughter and sale of meat have changed over the past 5-10 years, how? What do you think has caused these changes?
4. How would you, as a [job title], know if an animal/meat is not suitable for human consumption? (Signs, smells, etc.)?
5. In the past 5-10 years, what new policies have been introduced and which policies have improved?

## B. Challenges in securing food safety of meat

1. In relation to animal-human disease, what kinds of places/things do you inspect and what do you look for?
2. What difficulties do you face in making sure meat is safe to eat?
3. In your job as [job title], have you had any successes at identifying or preventing animal-to-human disease? What were these?
4. What do you do when you find meat/animals unsuitable for human consumption? (What did they do when faced with evidence of disease? What are they able to do and what can they not do?

## C. Policies and regulations helping/hindering

1. Do you make reports? If so, who do you report to? What do you report? How do you report (phone, written report, sent by bus)? Do you think it works?
2. Do you have specific legislation/policies that you have to enforce? What are these?
3. In your work, are there specific policies that help you, or make it difficult for you, to ensure that diseases don’t pass from animals to humans? Can you explain more?
4. How has decentralisation-by-devolution affect your work?

## D. Shocks re food safety of meat

1. In all the years that you have been involved in this work, have you ever had any major events or problems with diseases? What were these? How did this affect your role and relationships?
2. Do you think there will be any major problems regarding diseases passing from animals to humans in the future? What will these be and what will cause them?
3. Do you think there is more that can be done to control animal diseases and keep people safe? Who should take responsibility for this?
4. What do you think of the Abattoir in Arusha as a means to slaughter animals and control disease?
5. Would you like to make any other comments?
